# Analyses of the combination of 6-MP and dasatinib in cell culture

**DOI:** 10.3892/ijo.2013.1930

**Published:** 2013-05-02

**Authors:** GURMEET KAUR, HOLGER BEHRSING, RALPH E. PARCHMENT, MYRTLE DAVIS MILLIN, BEVERLY A. TEICHER

**Affiliations:** 1 Molecular Pharmacology Branch, Developmental Therapeutics Program, Division of Cancer Treatment and Diagnosis, Frederick, MD 21702;; 2 Laboratory for Investigative Toxicology, Applied/Developmental Research Directorate, SAIC-Frederick Inc., Frederick National Laboratory for Cancer Research, Frederick, MD 21702;; 3 Toxicology and Pharmacology Branch, National Cancer Institute, Bethesda, MD 20852, USA; 4 Molecular Pharmacology Branch, Developmental Therapeutics Program, Division of Cancer Treatment and Diagnosis, National Cancer Institute, Bethesda, MD 20852, USA

**Keywords:** 6-mercaptopurine, dasatinib, combination index, isobologram, response surface

## Abstract

A major tenet of cancer therapeutics is that combinations of anticancer agents with different mechanisms of action and different toxicities may be effective treatment regimens. Evaluation of additivity/synergy in cell culture may be used to identify drug combination opportunities and to assess risk of additive/synergistic toxicity. The combination of 6-mercaptopurine and dasatinib was assessed for additivity/synergy using the combination index (CI) method and a response surface method in six human tumor cell lines including MCF-7 and MDA-MB-468 breast cancer, NCI-H23 and NCI-H460 non-small cell lung cancer, and A498 and 786-O renal cell cancer, based on two experimental end-points: ATP content and colony formation. Clonal colony formation by human bone marrow CFU-GM was used to assess risk of enhanced toxicity. The concentration ranges tested for each drug were selected to encompass the clinical C_max_ concentrations. The combination regimens were found to be additive to sub-additive by both methods of data analysis, but synergy was not detected. The non-small cell lung cancer cell lines were the most responsive among the tumor lines tested and the renal cell carcinoma lines were the least responsive. The bone marrows CFU-GM were more sensitive to the combination regimens than were the tumor cell lines. Based upon these data, it appears that the possibility of enhanced efficacy from combining 6-mercaptopurine (6-MP) and dasatinib would be associated with increased risk of severe bone marrow toxicity, so the combination is unlikely to provide a therapeutic advantage for treating solid tumor patients where adequate bone marrow function must be preserved.

## Introduction

One of the main tenets of cancer therapeutics is that combinations of anticancer agents with different targets or different mechanisms of action and varied toxicities will produce better therapeutic outcomes. The premise of controlled randomized trials is that there is uncertainty whether any combination of treatments may have more benefit than the individual single treatments. Indeed, very large studies analyzing outcomes from multiple clinical trials most often find no substantive differences between the combination treatment regimen and the single agent arms lending support to the continuing need for randomized trials (1,2).

Objective mathematical and graphical methods for the assessment of additivity, synergy, and antagonism have been defined, including combination index, median effect, isobolograms, continuous measures, Bliss methodology and varied response surface techniques (3–17). Drug-drug interactions are inherently defined by a 3-dimensional (3D) concentration- or dose-response surface (17). 3D methods have several advantages: i) the response surface can be directly visualized and plotted; ii) predicted additive effects can be calculated using either the similar site or dissimilar site assumptions of additivity and the additive surface can be subtracted from the experimental surface to highlight areas of synergy and antagonism; iii) the synergy and antagonism can be quantified allowing varied drug combinations to be compared; and iv) the data can be analyzed for statistical significance. 3D analysis highlights stoichiometric or other relationships which may elucidate mechanisms of synergy. These methods can be effectively applied to cell-based and *in vivo* preclinical data.

Predicting from preclinical studies whether a potential new anticancer agent will have a positive therapeutic index in patients remains a challenge. The mouse is the traditional preclinical host for anticancer compound testing. Although the mouse is often a good predictor for certain organ system toxicities and mechanism of action, there are species differences. Bone marrow is critically sensitive to many antineoplastic agents, and combinations of agents with overlapping target organ toxicity may increase the risk of additive bone marrow toxicity (18). Mouse bone marrow is often less sensitive to cytotoxic agents than human bone marrow, resulting in exposures used during preclinical efficacy testing that cannot be achieved in patients (18–22). Bone marrow granulocyte macrophage-colony forming unit (CFU-GM) assays comparing the sensitivity of bone marrow cells across species are useful for predicting the blood levels of an agent that might be achieved in patients relative to those achievable in preclinical efficacy and safety species. Drug combinations with small or no differential in bone marrow progenitor sensitivity between species may have a better potential for reaching the efficacious exposure level of mice in patients, when bone marrow toxicity is dose limiting. It has been suggested that the ratio of mouse/human CFU-GM IC_90_ values equals the ratio of maximum tolerated doses in mouse and man for myelosuppressive agents, so the human maximum tolerated dose of an experimental compound could be predicted and thus the potential for achieving a therapeutic blood level in patients estimated prior to clinical development (18).

6-Mercaptopurine (6-MP) was synthesized and developed by Hitchings and Elion in the 1950s as one of a large series of purine analogs designed to interfere with nucleic acid biosynthesis. 6-MP is active against human leukemia (23). Monitoring plasma 6-MP after an oral dose is of questionable value due to high inter-patient variability in plasma levels. 6-MP moves rapidly into the anabolic and catabolic pathways for purines. The active intracellular metabolites have longer half-lives than the parent drug. The biochemical effects of a single 6-MP dose are evident long after the parent drug has disappeared from plasma (24). 6-MP competes with hypoxanthine and guanine for the enzyme hypoxanthine-guanine phosphoribosyltransferase (25). 6-MP is metabolized to thioinosinic acid. Thioinosinic acid inhibits several reactions involving inosinic acid, including the conversion of inosinic acid to xanthylic acid and to adenylic acid via adenylosuccinate. 6-Methylthioinosinate is formed by the methylation of thioinosinic acid. Both thioinosinic acid and methylthioinosinic acid inhibit the first enzyme in the *de novo* purine ribonucleotide synthesis pathway. 6-MP is found in DNA in the form of deoxythioguanosine. Some 6-MP is converted to nucleotide derivatives of 6-thioguanine (6TG) by the sequential actions of inosinate dehydrogenase and xanthylate aminase, converting thioinosinic acid to thioguanylic acid. Preclinical tumors resistant to 6-MP often cannot convert 6-MP to thioinosinic acid (26,27). However, many mechanisms of resistance to 6-MP have been identified, particularly in human leukemias (28). It is not known which biochemical effect of 6-MP and its metabolites are predominantly responsible for cell death. Bone marrow suppression is a 6-MP dose-limiting toxicity and may be more profound when 6-MP is administered with other myelosuppressive agents.

Deregulated BCR-ABL tyrosine kinase activity is the molecular marker for chronic myeloid leukemia (CML). Imatinib, a BCR-ABL TK inhibitor, is the front-line therapy for CML. However, patients develop resistance to imatinib with up to 90% of patients in the accelerated/blastic phase resistant. Based on modeling studies, dasatinib was predicted to bind to multiple conformations of the ABL kinase, and it can produce durable responses in patients with many BCR-ABL mutations highly resistant to imatinib. Dasatinib is recommended for CML in chronic, blastic or accelerated phase that is resistant to imatinib (29). Dasatinib inhibits BCR-ABL, SRC family (SRC, LCK, YES, FYN), c-KIT, EPHA2, and PDGFRβ at nanomolar concentrations, and likely inhibits the activity of upregulated c-Abl following genotoxic agents or γ-irradiation (30). Dasatinib overcomes imatinib resistance resulting from BCR-ABL kinase domain mutations, activation of alternate SRC family kinase signaling pathways (LYN, HCK), and multi-drug resistance gene overexpression (31). The cellular effects of dasatinib are widespread and not limited to immediate BCR-ABL targets affecting downstream MAPK pathways (32). Dasatinib interacts with many proteins involved in processing and repair of DNA damage such as p53, p73, Mdm2, Rad51, DNA-PK, WRN, CSB and BRCA1. Dasatinib induces myelosuppression in leukemia patients and is the most common reason for dose reduction. Data suggest that dasatinib may increase the severity and frequency of myelosuppression when given in combination with agents with myelosuppressive effects (33).

The current cell-based study explored the combination of 6-MP and dasatinib in 6 human tumor cells lines using two experimental end-points and two methods for determination of additivity/synergy. The colony formation end-point for the tumor cell lines is compared with colony formation by human bone marrow CFU-GM exposed to the drug combination.

## Materials and methods

### Materials

6-Mercaptopurine (NSC755) and dasatinib (NSC732517) were obtained from the DTP compound repository. Both compounds were formulated as 50 mM stock solutions in DMSO (Sigma-Aldrich, St. Louis, MO, USA), aliquoted, stored at −70°C and diluted with RPMI-1640 medium to the appropriate concentrations for experiments. For CFU-GM experiments, both compounds were formulated in DMSO as 4,000X target concentration stock solutions.

### Cell lines and culture

All cell lines were purchased from ATCC (Manassas, VA, USA). The MCF-7 breast adenocarcinoma line was established from pleural effusion of a 69-year-old female patient in the early 1970s (34,35). MCF-7 cells are ER^+^ and p53 wild-type (36). MDA-MB-468 breast adenocarcinoma line was established from a 51-year-old female patient in the 1970s and is ER^+^ and p53 mutant (37,38). The NCI-H23 lung adenocarcinoma cell line was established from a 51-year-old male patient in the 1970s and has mutant K-ras, mutant p53 and has c-myc gene amplification (39,40). The NCI-H460 lung large cell carcinoma was developed from the pleural effusion of a male patient in 1982 (41). The NCI-H460 cell line has wild-type p53 (42). The A498 renal cell carcinoma line was established from the kidney cancer of a 520year-old patient in the early 1970s and has wild-type p53 (43,44). The 786-O renal cell adenocarcinoma cell line was established from the primary clear cell adenocarcinoma of a 58-year-old male in the early 1970s and has mutant p53 (45,46). All of the cell lines were maintained in RPMI-1640 medium (Life Technologies, Grand Island, NY, USA) supplemented with 5% fetal bovine serum (HyClone/Thermo Fisher Scientific, Logan, UT, USA) and glutaMAX™ (Life Technologies, Grand Island, NY, USA) in a humidified 5% carbon dioxide atmosphere at 37°C.

### Growth inhibition assay

Six human tumor cell lines were exposed to a concentration range of 6-MP, dasatinib or combinations of 6-MP and dasatinib in 3 to 4 independent experiments. Cells were plated in 96-well tissue culture plates in 100 *μ*l RPMI medium supplemented with 5% FBS and glutamine at different cell seeding densities depending upon the properties of the cell line: the initial seeding densities were: MCF-7, 5×10^3^; MDA-MB-468, 2.5×10^3^; NCI-H23, 2.5×10^3^; NCI-H460, 1×10^3^; A498, 1.25×10^3^ and 786-O, 1.25×10^3^ cells/well. Eight concentrations of 6-MP (0.03 to 100 *μ*M) or dasatinib (0.001 to 3 *μ*M) in half-log intervals were tested. Plates were incubated overnight at 37°C in humidified air with 5% CO_2_ prior to the addition of 6-MP or dasatinib for a 72-hour drug exposure at 37°C with humidified air/5% CO_2_. After the incubation period, the test plates were allowed to stand at room temperature for 10 min; 100 *μ*l of media was removed from each well and replaced with 100 *μ*l of CellTiter-Glo^®^ (Promega, Madison, WI, USA) at room temperature according to the manufacturer’s instructions. The plates were allowed to stand at room temperature for 30 min and luminescence was read on Infinity 200M (Tecan Systems Inc, Grödig, Austria). Luminescence data were converted to growth fraction by comparison with the luminescence for the untreated control for each cell line, and IC_50_ and IC_90_ values determined from the graphical data. Each cell line was tested in at least 3 independent experiments.

### Colony formation assay

Each of the 6 cell lines were grown as monolayers in RPMI-1640 medium supplemented with 5% FBS and glutamine in 6-well dishes. The 6-MP and dasatinib were tested over a concentration range from centering on the clinical achievable circulating C_max_ for each agent alone and in combination. Cultures were exposed to the compounds for 3 days at 37°C in a humidified atmosphere of 5% CO_2_. The cells were suspended by exposure to trypsin then plated for colony formation in 6-well dishes in different numbers depending upon the properties of the cell line. The cells were plated in 6-well plates in a RPMI-1640 medium supplemented with 5% FBS and glutamine: MCF-7, 1.2×10^3^; MDA-MB-468, 1.2×10^3^; NCI-H23, 1×10^3^; NCI-H460, 0.75×10^3^; A498, 0.75×10^3^ and 786-O, 0.75×10^3^. Each treatment group was tested in triplicate wells. Each experiment was conducted at 2 independent times. After 7 to 12 days, colonies were fixed and stained with 0.5% w/v crystal violet in 20% methanol. Colonies were defined as clusters containing 50 or more cells. Colonies were counted using a GelCount (Oxford Optronix, Oxford, UK). The IC_50_ and IC_90_ values were determined from the graphical data.

### Human bone marrow

For CFU-GM, the assay was conducted using freshly collected, human bone marrow mononuclear cells (Lonza-Biowhittaker, Walkersville, MD, USA).

### Bone marrow CFU-GM assay

The semi-solid matrix agarose based CFU-GM assay was used to establish levels of toxicity for the compounds tested. Human bone marrow cells were received from the vendor on ice and upon arrival, the cells were gently pelleted and the transport media removed. The cells were then suspended in 5 ml of Plasma-Lyte A USP (Baxter Healthcare, Deerfield, IL, USA), mixed well and treated with 2.5 *μ*l/ml Pulmozyme (Genentech Inc., South San Francisco, CA, USA). After 10 min at room temperature, the cells were layered over 5 ml Ficoll-Paque PLUS (Stem Cell Technologies, Vancouver, BC, Canada) and centrifuged for 30 min at 1,500 × g relative centrifugal force to enrich the viable mononuclear cell population. The buffy layer containing the mononuclear cells (MNC’s) was collected, washed in 14 ml Plasma-Lyte A USP, and finally suspended in 10 ml IMDM (Stem Cell Technologies). Cell counts were performed using Beckman Coulter ViCell cell counter. A minimal volume of cell suspension was added to 3.5 ml complete medium containing IMDM, 20% fetal bovine serum (Lonza, Walkersville, MD, USA), 100 ng/ml gentamicin (Abraxis, Schaumburg, IL, USA), and 10 ng/ml Leukine sargramostim rhGM-CSF (Berlex, Seattle, WA, USA) in 15 ml conical tubes. For each test concentration, drugs were solubilized in DMSO at 4,000-fold stock solution concentration. To create the drug combination stock solutions relevant drug stocks (or drug stock + DMSO for single agent control groups) were mixed together in a 1:1 ratio to form 2,000X stock solutions. A 5 *μ*l aliquot of drug stock solution was added into 5 ml of complete medium containing 1.3X FBS, gentamicin and rhGM-CSF in a 15 ml conical tube and mixed and this 1,000X diluted drug stock solution in medium was then transferred to the 3.5 ml cell suspension and mixed. After warming, 1.5 ml of 2.5% SeaPlaque Agarose (Lonza-Biowhittaker, Walkersville, MD, USA; catalog # 50101) in water was added, mixed using a vortex mixer, and 2 ml was plated in triplicate in 6-well plates containing a pre-gelled, 2 ml under-layer of IMDM, FBS and 2.5% SeaPlaque Agarose per well. For 72-h pulse exposures, the 1.5 ml agarose solution was substituted with medium and the entire 10 ml contents were transferred to 25 cm^2^ vented cap, canted neck culture flasks (Corning, Manassas, VA, catalog # 430639) for incubation until the completion of the 72-h period. At the end of the 72-h period, the entire content of the flasks were transferred to individual 15 ml conical tubes and the flask rinsed with an additional 3 ml medium that was added to the respective tube of the flask. The tubes were gently spun (190X G) for 5 min to pellet the cells and treatment + rinse medium removed. The contents of each flask were reconstituted in 8.5 ml medium, 1.5 ml warmed agarose solution was added and cell suspensions were plated as done for the constant exposure group. For constant and pulse exposure groups, each well contained 2×10^5^ human donor MNC and total culture time (including the 72-h exposure period) was 14 days. The plates were maintained at 4°C until completely gelled (usually 15–20 min) and then placed in a humidified incubator at 37°C with 5% CO_2_. After 14 days, colonies >64 cells were counted manually, and the treatment effect calculated from the reduction in colonies per well as percent of vehicle control.

### Data analysis

Data analysis for additivity was performed using the MacSynergy II program (Prichard and Shipman, University of MI, Ann Arbor, MI) and CompuSyn program (Chou and Martin). CompuSyn program (Chou and Martin) was used to compute a combination index (CI) for drug combinations studied with growth assays and colony formation assays. The Chou-Talalay combination-index method for drug combination is based on the median-effect equation, derived from the mass-action law principle, which is the unified theory that provides the common link between single entity and multiple entities, and first order and higher order dynamics. This general equation encompasses the Michaelis-Menten, Hill, Henderson-Hasselbalch and Scatchard equations in biochemistry and biophysics. The resulting combination index (CI) theorem of Chou-Talalay offers quantitative definition for additive effect (CI=1), synergism (CI<1) and antagonism (CI>1) in drug combinations. This theory also provides algorithms for computer simulation of synergism and/or antagonism at any effect and concentration/dose level, as shown in the CI plot and isobologram, respectively (14,47).

The MacSynergy II program calculates the theoretical additive interactions of the drugs based on the Bliss Independence mathematical definition of expected effects for drug-drug interactions. The Bliss Independence model is based on statistical probability and assumes that the drugs act independently. MacSynergy II provides a 3D model for additivity analysis of drug combinations and contour plot. The calculated theoretical additive interactions are determined from the concentration response data of the individual drugs. The calculated additive surface, which represents the predicted additive interaction, is then subtracted from the observed surface to show regions of greater-than-expected (synergy) or less-than-expected (antagonism) interactions. If the interactions are additive, the resulting surface appeared as a horizontal plane at 0% above the calculated additive surface in the resulting difference plots. Peaks above this plane in the difference plots are indicative of synergy, while depressions below the horizontal plane indicate antagonism (17).

## Results

**Figure 1.**
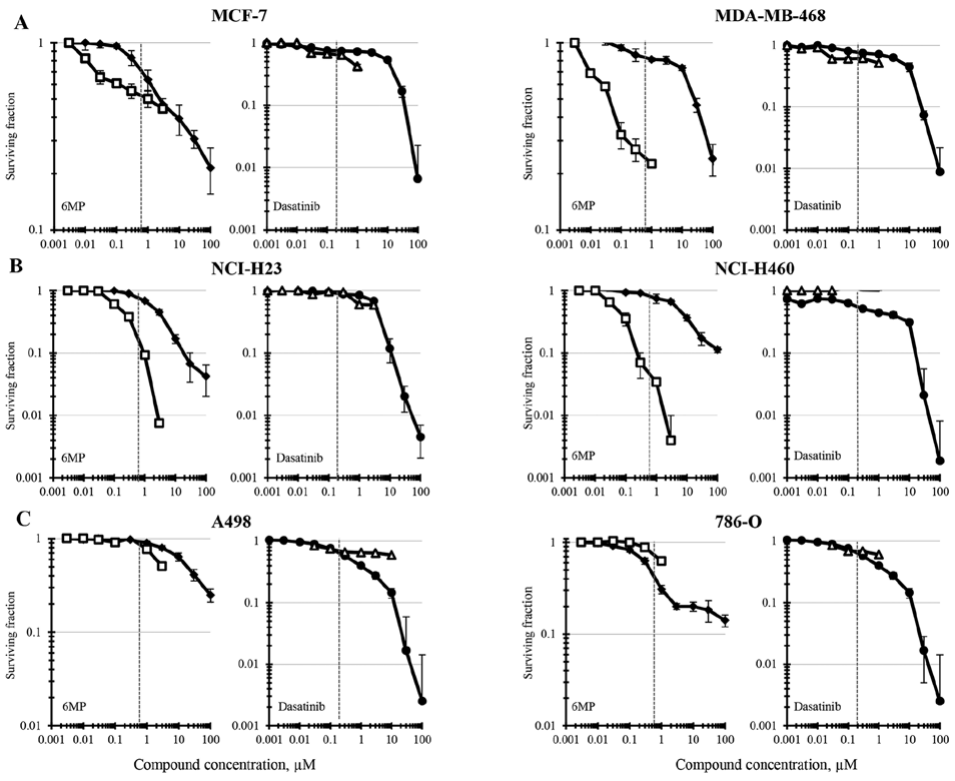
(A) Concentration response for the MCF-7 and MDA-MB-468 human breast carcinoma cell lines upon exposure to 6-MP or dasatinib using ATP content (CellTiter-Glo) (solid symbols) or colony formation (open symbols) as the end-points. (B) Concentration response for the NCI-H23 and NCI-H460 human non-small cell lung carcinoma cell lines upon exposure to 6-MP or dasatinib using ATP content (CellTiter-Glo) (solid symbols) or colony formation (open symbols) as the end-points. (C) Concentration response for the A498 and 786-O human renal cell carcinoma cell lines upon exposure to 6-MP or dasatinib using ATP content (CellTiter-Glo) (solid symbols) or colony formation (open symbols) as the end-points. The vertical dotted lines are at the clinical Cmax concentrations for each drug. The data are the means of 2–4 independent experiments; error bars, SD.

Six human tumor cell lines, 2 breast cancer, 2 non-small cell lung cancer and 2 renal cell carcinoma, were selected for study. The compounds were tested as single agents in each cell line using ATP content (CellTiter-Glo luminescence) and colony formation as end-points (Fig. 1). Concentrations of 6-MP and dasatinib were selected to cover several logs encompassing the clinical achievable C_max_ for both assays. The reported clinical C_max_ for 6-MP is 0.6 *μ*M and the clinical C_max_ for dasatinib is reported to be 0.2 *μ*M (48,49). The 6-MP IC_50_ concentrations in the MCF-7 human breast carcinoma cell line were similar for both experimental end-points and were 1–2 *μ*M (Fig. 1A). In the same cell line, dasatinib had an IC_50_ of 11 *μ*M by ATP content and of 0.6 *μ*M by colony formation. The MDA-MB-468 breast carcinoma line was less sensitive to 6-MP as determined by ATP content, having an IC_50_ of 23 *μ*M but more sensitive by the cell survival measurement of colony formation with an IC_50_ of 0.04 *μ*M. The dasatinib IC_50_ in MDA-MB-468 cells determined by ATP content was 7.5 *μ*M and by colony formation was 0.7 *μ*M. Both the NCI-H23 and the NCI-H460 non-small cell lung carcinoma cell line were more sensitive to 6-MP when survival was measured by colony formation than was determined by ATP content (Fig. 1B). The 6-MP IC_50_s for the NCI-H23 and NCI-H460 cells determined by colony formation were 0.15 and 0.06 *μ*M and by ATP content were 2.6 and 8 *μ*M, respectively. The non-small cell lung carcinoma cell lines had differing sensitivity to dasatinib having IC_50_s of 4.5 and 0.3 *μ*M as determined by ATP content for the NCI-H23 and NCI-H460 lines, respectively. However, neither the NCI-H23 line nor the NCI-H460 cells were responsive to dasatinib in the concentration range tested by colony formation (<50% reduction). The 6-MP IC_50_ for the A498 renal cell carcinoma line was 24 *μ*M as determined by ATP content and 2.8 *μ*M as determined by colony formation (Fig. 1C). The dasatinib IC_50_ in the same cell line was 0.6 *μ*M as determined by ATP content and >20 *μ*M as determined by colony formation. Finally, the 6-MP IC_50_ for the 786-O renal cell carcinoma cell line was 0.45 *μ*M as determined by ATP content and >1.25 *μ*M as determined by colony formation. For the 786-O line, the dasatinib IC_50_ was 0.5 *μ*M when determined by ATP content and >1 *μ*M when survival was determined by colony formation.

**Figure 2.**
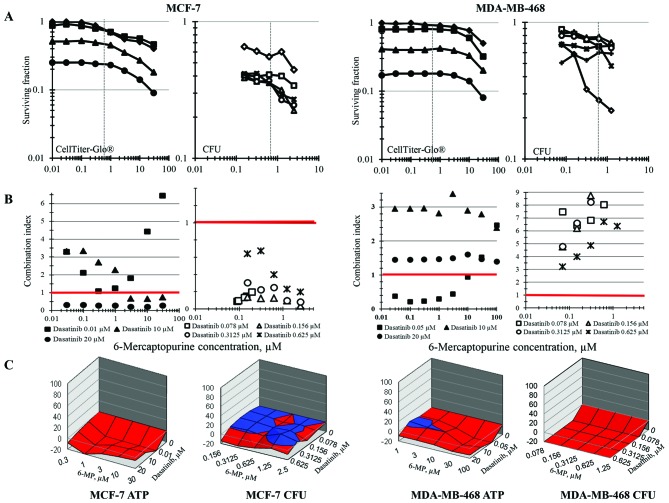
(A) Concentration response for the MCF-7 and MDA-MB-468 human breast carcinoma cell lines upon exposure to simultaneous combinations of 6-MP and dasatinib using ATP content (CellTiter-Glo) (solid symbols) or colony formation (open symbols) as the end-points. (B) Combination index data analysis of the concentration response data in A panels. (C) Response surface data analysis of the concentration response data in A panels. Red surface area indicates less-than-additivity and blue surface area indicates additivity. The vertical dotted lines in panels A and B are at the clinical Cmax concentrations for 6-MP. The data are the means of 2–4 independent experiments; error bars, SD.

Simultaneous combinations of 6-MP and dasatinib were assessed in the same 6 human tumor cell lines by ATP content and colony formation and analyzed for additivity/synergy using combination index methodology and response surface methodology. The concentration ranges selected for the combination studies encompassed the clinical C_max_ concentrations for each drug. Using ATP content as an end-point, the response curves for 6-MP with increasing concentrations of dasatinib tend to be parallel except at the highest concentrations indicating that the drugs are not interacting. In the MCF-7 breast carcinoma cell line, the combinations of 6-MP and dasatinib were sub-additive as determined by ATP content using the combination index method except at the very high dasatinib concentration of 20 *μ*M and were sub-additive across all concentration combinations by the response surface area method. However, when MCF-7 cell survival was measured by colony formation, all of the combinations of 6-MP and dasatinib produced greater than additive cell killing by the combination index data analysis method and were additive to sub-additive by the response surface area method (Fig. 2). As determined by ATP content, combinations of 6-MP and dasatinib were sub-additive to additive both by combination index analysis and response surface are analysis in the MDA-MB-468 breast carcinoma cell line. On the other hand, using the colony formation end-point, both methods of data analysis indicate that the combinations were sub-additive across all combinations (Fig. 2).

**Figure 3.**
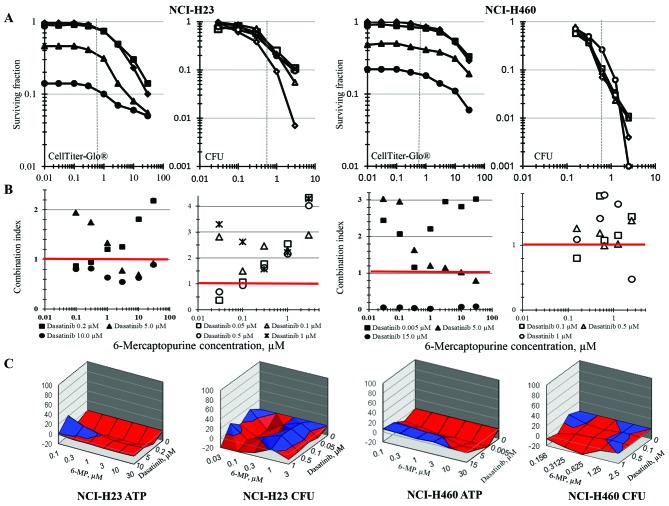
(A) Concentration response for the NCI-H23 and NCI-H460 human non-small cell lung carcinoma cell lines upon exposure to simultaneous combinations of 6-MP and dasatinib using ATP content (CellTiter-Glo) (solid symbols) or colony formation (open symbols) as the end-points. (B) Combination index data analysis of the concentration response data in A panels. (C) Response surface data analysis of the concentration response data in A panels. Red surface area indicates less-than-additivity and blue surface area indicates additivity. The vertical dotted lines in panels A and B are at the clinical Cmax concentrations for 6-MP. The data are the means of 2–4 independent experiments; error bars, SD.

Experiments testing the simultaneous combination of 6-MP and dasatinib in the NCI-H23 and NCI-H460 non-small cell lung carcinoma cell lines examined a wide dasatinib concentration range with the ATP content end-point and a narrower dasatinib concentration range centered on the clinically achievable dasatinib C_max_ concentration with the colony formation end-point (Fig. 3). The combination index for 6-MP and dasatinib in the NCI-H23 non-small cell lung cancer line found modest synergy at the very high dasatinib concentration of 10 *μ*M and sub-additivity at lower dasatinib concentrations, while the response surface analysis indicated sub-additive to additive response to the combination regimens. Similarly, by colony formation, the combination index analysis and response surface analysis found the combination of 6-MP and dasatinib to be additive to sub-additive. In the NCI-H460 cell line, dasatinib at the very high concentration of 15 *μ*M was greater than additive when combined with 6-MP in the ATP content assay using the combination index analysis method; however, the response surface method found the combinations to be primarily sub-additive. In the NCI-H460 cells, the colony formation survival assay found the 6-MP and dasatinib combination was primarily sub-additive by both the combination index and response surface methods of data analysis.

**Figure 4.**
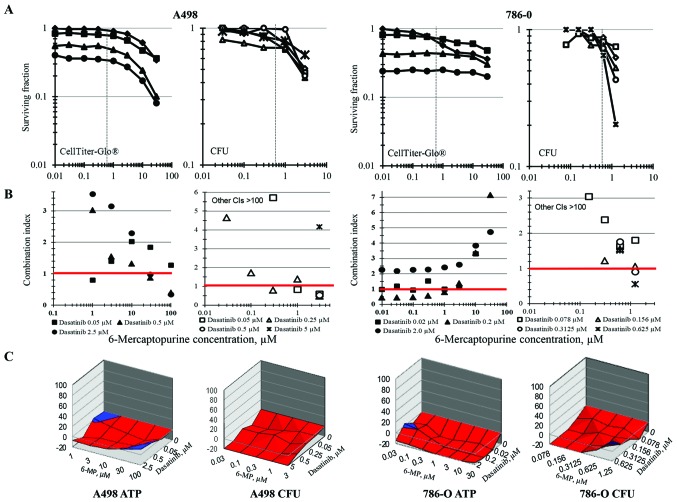
(A) Concentration response for the A498 and 786-O human renal cell carcinoma cell lines upon exposure to simultaneous combinations of 6-MP and dasatinib using ATP content (CellTiter-Glo) (solid symbols) or colony formation (open symbols) as the end-points. (B) Combination index data analysis of the concentration response data in A panels. (C) Response surface data analysis of the concentration response data in A panels. Red surface area indicates less-than-additivity and blue surface area indicates additivity. The vertical dotted lines in panels A and B are at the clinical Cmax concentrations for 6-MP. The data are the means of 2–4 independent experiments; error bars, SD.

The renal cell carcinoma cell lines A498 and 786-O had a primarily sub-additive response to the combination of 6-MP and dasatinib as determined by ATP content using the combination index method of data analysis (Fig. 4). Interestingly, when 6-MP and dasatinib were tested in the renal cell carcinoma line by colony formation to measure survival, data analysis by the combination index method indicated that the combination was additive to antagonistic producing combination index values >100 for most of the concentrations tested. The response surface data analysis similarly showed sub-additive response to the combinations regimens over all concentrations tested.

**Figure 5.**
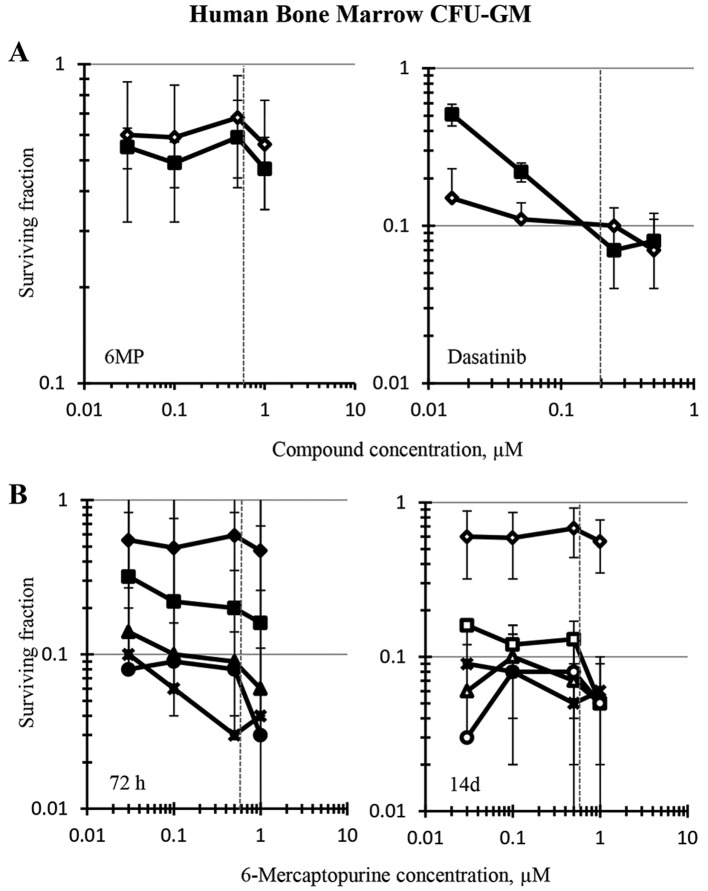
(A) Concentration response for human bone marrow CFU-GM upon exposure to 6-MP or dasatinib using colony formation as the end-point. The cells were exposed to each drug for 72 h (solid symbols) or continuously for 14 days (open symbols). (B) Concentration response for human bone marrow CFU-GM upon exposure to combinations of 6-MP and dasatinib using colony formation as the end-point. The cells were exposed to the combination for 72 h (solid symbols) or continuously for 14 days (open symbols). The symbols indicate concentrations of dasatinib: 6-MP alone (♦, ⋄); 0.015 *μ*M (▪, □); 0.05 *μ*M (▴, ▵), 0.25 *μ*M (•, ○) and 0.5 *μ*M (*). The vertical dotted lines are at the clinical C_max_ concentrations for each drug. The data are the means of 2–4 independent experiments; error bars are SD.

Human bone marrow CFU-GM was modestly sensitive to 6-MP upon 72-h exposure or 14-day continuous exposure in a concentration range of drug that centered on its clinical C_max_ of 0.6 *μ*M, reaching an IC_50_ at 1 *μ*M, the highest concentration tested (Fig. 5A). Dasatinib was a more potent cytotoxicant than 6-MP to human bone marrow CFU-GM and exhibited a concentration X time response to the drug. The 14-day continuous exposure to dasatinib produced greater inhibition of colony formation than 72-h exposure at concentrations below its clinical C_max_ of 0.2 *μ*M (Fig. 5A). The IC_90_ for 72-h exposure to dasatinib was 0.15 *μ*M, whereas huCFU-GM colony formation was inhibited 90% by continuous exposure to 0.05 *μ*M dasatinib (Fig. 5, note standard deviation at this concentration). The 6-MP and dasatinib combination was cytotoxic to the bone marrow CFU-GM upon both 72-h and 14-day exposure. Clinically-relevant dasatinib concentrations of 0.05 to 0.25 *μ*M combined with the clinical C_max_ concentration of 6-MP (0.6 *μ*M) inhibited huCFU-GM by 90% or more using either 72-h or 14-day exposures (Fig. 5B). Comparing the CFU-GM toxicity of dasatinib alone and in combination with 6-MP indicated that the huCFU-GM toxicity of dasatinib dominated that of 6-MP.

## Discussion

Both 6-MP and dasatinib are used for the treatment of human leukemia. 6-MP and dasatinib have different mechanisms of action and by inhibiting differing pathways in cancer cells, are hypothesized to have greater-than-additive cytotoxicity in combination. The thiopurines azathioprine, 6-thioguanine (6TG) and 6-MP are effective anti-inflammatory, anticancer and immunosuppressive drugs and have been in clinical use for over half a century. 6-MP and azathioprine received FDA approval in 1953 and 1968, respectively (50,51). Dasatinib was approved by the FDA in 2006 for the treatment of resistant, recurrent chronic myelogenous leukemia, based upon potent inhibition of several mutant forms of the BCR-ABL kinase that leads to improved survival. As evidenced by concentration response curves, dasatinib may have off-target effects at higher than clinically achievable concentrations. It potently inhibits other kinases including NEK2 and CLK2. NEK2, NIMA (never in mitosis A)-related kinase, is a homodimeric serine/threonine kinase that localizes to centrosomes at the onset of mitosis. NEK2 phosphorylates the intercentrosome linker proteins, thereby disconnecting the centrosomes and allowing separation (52). Inhibition of NEK2 would impair chromosome segregation. CLK2 is a member of the cdc2-like kinase family which functions by phosphorylating the spliceosome serine-arginine proteins within the spliceosome assembly, thus facilitating alternate splicing of pre-mRNAs into protein-encoding mRNAs leading to protein diversity. Inhibition of CLK2 would produce misregulation of pre-mRNA splicing (53).

There are several mechanisms by which chemotherapy combinations can produce metabolic imbalance in cells leading to cell death; sequential inhibition of multiple enzymes in the same pathway, concurrent blockade of multiple pathways leading to the same critical end-product or complimentary inhibition of multiple pathways in a critical metabolic process (54). Additionally, the term ‘horizontal combination’ describes combining inhibitors of different pathways in two or more cell types involved in malignant disease, and the term ‘vertical combination’ describes combining inhibitors of the same or related pathways in two or more cell types involved in malignant disease (55).

The current study evaluated the simultaneous combination of 6-MP and dasatinib in six human tumor cell lines and in human bone marrow CFU-GM, a hematopoietic progenitor of the neutrophil lineage. Using the 72-h ATP content end-point assay, the clinically achievable concentrations of single agent 6-MP reached 50% response only in the 786-O renal cell carcinoma line. However, in the colony formation survival assay, a 72-h exposure to 6-MP achieved 50% cell kill in both the MCF-7 and MDA-MB-468 breast carcinoma cell lines, and 90% cell kill was achieved at the 6-MP clinical C_max_ concentration in both the NCI-H23 and NCI-H460 non-small cell lung carcinoma cell lines. Using the 72-h ATP-content end-point, the clinical C_max_ concentration of single agent dasatinib reached 50% response only in the 786-O renal cell carcinoma line. However, by colony formation assays following a 72-h exposure to 6-MP, none of the cell lines reached 50% cell kill at or below the dasatinib C_max_ concentration.

The most striking difference in the combination data occurred with colony formation in the breast cancer cell lines: the 6-MP plus dasatinib combination was additive to greater than additive by response surface analysis and greater than additive by combination index analysis in the MCF-7 line, and markedly sub-additive by both analytical methods in the MDA-MB-468 line. Overall, there was reasonable agreement between the ATP content end-point and the colony formation end-point, when estimating combinations of concentrations achieving one-log cell kill and when analyzing for drug interaction with the combination index method or the response surface method. Among the tumor cell lines, the renal cell carcinoma lines were most resistant to the 6-MP plus dasatinib combination, while the non-small lung cancer lines were most responsive. However, the combination regimen was much more cytotoxic to bone marrow CFU-GM than to any of the tumor cell lines, exhibiting greater than one-log kill of these hematopoietic progenitors at the clinical C_max_ of 6-MP combined with dasatinib concentrations below its clinical C_max_. Because a 1-log reduction in marrow CFU-GM progenitors associates with maximum tolerated doses that cause severe neutropenia (18,21), these *in vitro* results suggest that dose reductions of 6-MP and/or dasatinib would likely be required to avoid severe myelosuppression in solid tumor patients where adequate marrow function needs to be maintained. In conclusion, although a scientific rationale can be described for the combination of 6-MP and dasatinib, it is unlikely that combining these two drugs will provide increased benefit to solid tumor patients.
